# Advancing UK Regulatory Science Strategy in the Context of Global Regulation: a Stakeholder Survey

**DOI:** 10.1007/s43441-021-00263-2

**Published:** 2021-02-16

**Authors:** Samantha Cruz Rivera, Barbara Torlinska, Eliot Marston, Alastair K. Denniston, Kathy Oliver, Steve Hoare, Melanie J. Calvert

**Affiliations:** 1 Birmingham Health Partners Centre for Regulatory Science and Innovation, University of Birmingham, Birmingham, UK; 2 Centre for Patient Reported Outcomes Research, Institute of Applied Health Research, College of Medical and Dental Sciences, University of Birmingham, Birmingham, UK; 3 Institute of Applied Health Research, College of Medical and Dental Sciences, University of Birmingham, Birmingham, UK; 4 Health Data Research, London, UK; 5 University Hospitals Birmingham NHSFT, Birmingham, UK; 6 Regulatory Horizons Council, London, UK; 7 International Brain Tumour Alliance (IBTA), Tadworth, UK; 8 The Association of the British Pharmaceutical Industry, London, UK; 9 National Institute for Health Research (NIHR) Birmingham Biomedical Research Centre, University of Birmingham, Birmingham, UK; 10 NIHR Surgical Reconstruction and Microbiology Research Centre, University Hospitals Birmingham NHS Foundation Trust, University of Birmingham, Birmingham, UK; 11 National Institute for Health Research (NIHR) Applied Research Centre West Midlands, University of Birmingham, Birmingham, UK

**Keywords:** Regulatory science, Health products, Medicines and devices

## Abstract

**Background:**

The UK’s transition from the European Union creates both an urgent need and key opportunity for the UK and its global collaborators to consider new approaches to the regulation of emerging technologies, underpinned by regulatory science. This survey aimed to identify the most accurate definition of regulatory science, to define strategic areas of the regulation of healthcare innovation which can be informed through regulatory science and to explore the training and infrastructure needed to advance UK and international regulatory science.

**Methods:**

A survey was distributed to UK healthcare professionals, academics, patients, health technology assessment agencies, ethicists and trade associations, as well as international regulators, pharmaceutical companies and small or medium enterprises which have expertise in regulatory science and in developing or applying regulation in healthcare. Subsequently, a descriptive quantitative analyses of survey results and directed thematic analysis of free-text comments were applied.

**Results:**

Priority areas for UK regulatory science identified by 145 participants included the following: flexibility: the capability of regulations to adapt to novel products and target patient outcomes; co-development: collaboration across sectors, e.g. patients, manufacturers, regulators, and educators working together to develop appropriate training for novel product deployment; responsiveness: the preparation of frameworks which enable timely innovation required by emerging events; speed: the rate at which new products can reach the market; reimbursement: developing effective tools to track and evaluate outcomes for “pay for performance” products; and education and professional development.

**Conclusions:**

The UK has a time-critical opportunity to establish its national and international strategy for regulatory science leadership by harnessing broader academic input, developing strategic cross-sector collaborations, incorporating patients’ experiences and perspectives, and investing in a skilled workforce.

**Supplementary Information:**

The online version of this article (10.1007/s43441-021-00263-2) contains supplementary material, which is available to authorised users.

## Introduction

The UK’s capability to adapt its regulatory frameworks in healthcare is under pressure. The pace of emerging technologies and the COVID-19 pandemic makes this a global issue. Furthermore, the UK leaving the European Union (EU) adds a significant, specific national need for change, carrying opportunity and risk that must be carefully managed. The scale and pace of innovation in existing and emerging technologies has outstripped the UK’s longstanding regulatory frameworks, and economic incentives need to be carefully counterbalanced by a key focus on patient safety, as well as evolving standards of evidence of efficacy. Regulatory science will be crucial to this endeavour.

Regulatory science in healthcare can be broadly defined as “the application of the biological, medical and sociological sciences to enhance the development and regulation of medicines and devices in order to meet the appropriate standards of quality, safety, and efficacy” [1]. However, there is no consensus definition, as different organisations such as US Food and Drug Administration (FDA) [2], the European Medicines Agency (EMA) [3] and the Pharmaceutical Society of Japan (PSJ) [4] have proposed their own definitions.

Regulatory frameworks face several parallel developments which undermine their effectiveness. Firstly, medical technologies, clinical practice and societal/public needs are rapidly evolving in fields such as digital devices, personalised medicine, health data and artificial intelligence (AI), as well as novel trial design and patient centricity in drug development [5–7]. These come with vast opportunities but also some significant risks to users, innovators, and payers [8]. However, through the development of regulatory and scientific strategy, planning and governance regulatory science can be future-proofed [9]. Secondly, post-Brexit, the UK intends to separate from EU regulations at the end of the transition period, 31 December 2020. Currently, the UK Government is planning to develop a regulatory framework for medicines and medical devices that will allow the fast approval of the most innovative and cost-effective products for the National Health Service (NHS) while maintaining a globally competitive and internationally collaborative position [10, 11]. Finally, the tremendous national and international efforts to tackle COVID-19 have highlighted both challenges and benefits of regulatory flexibility in rapid but effective support for new diagnostics and vaccines in response to the pandemic.

The importance and the scope of regulatory science may not always be fully recognised by academic, policy and clinical communities, whose focus may be either technological innovation or producing evidence on the safety, effectiveness and cost-effectiveness of interventions to inform regulators and policymakers, rather than the science of developing new tools, standards and approaches to underpin advances in regulation [12]. Hence, opportunities for successful collaboration can be missed between academic, clinical, pharmaceutical, health technology and regulatory scientists, as well with patients and the public themselves, whose ‘voice’ is essential in the development of healthcare interventions. Such lost opportunities may come with societal costs of delayed licensing—and thus delayed patient benefit—or conversely, underestimated risks of adverse effects of already licenced products and technologies.

To meet the challenges outlined above, a well-informed and well-coordinated UK environment for regulatory change in healthcare, underpinned by strong regulatory science, is required, which must leverage collaboration with academia, industry, patients, regulators and international stakeholders. This manuscript aimed to identify the most accurate definition of regulatory science, identify critical emerging challenges and opportunities in regulatory science, define strategic areas of regulation of healthcare innovation which can be enabled through regulatory science and explore training and infrastructure needed to advance UK and international regulatory science. The authors represent a consortium of stakeholders keen to raise the profile of regulatory science in the UK; the article aims to promote debate and drive action.

## Methods

An anonymised online survey (Appendix 1) was developed, containing questions on (1) demographics, (2) the definition of regulatory science in healthcare, (3) current challenges and opportunities for UK regulatory science, (4) strategic areas for development, (5) current training and future needs, and (6) infrastructure required. The ‘Current challenges and opportunities’ section was based upon ‘The Development of Regulatory Science in the UK: A Scoping Study’ [1] and the European Medicines Agency’s goals for regulatory science to 2025 [6]. Four of the main “Strategic areas for development” were extracted verbatim from the Medicines and Healthcare Products Regulatory Agency (MHRA) Corporate Plan 2018–2023 [12]. The MHRA Corporate Plan includes the strategic area “Public health and partnership” which was replaced in the survey with “Patient-centred drug development” to emphasise the crucial role of patients in the development of healthcare products.

The section “Current training and future needs” aimed to explore awareness and availability of the training, both within and outside of UK, barriers and enablers for the existing offer, and future training needs. Additionally, views on the focus and the level of education and training were sought. The questions on the existing and desirable collaborations and infrastructure were to identify the needs and to inform the role of academia within regulatory science.

Questions contained space for free-text comments, to allow respondents to expand on their answers or challenge the authors’ assumptions. The team decided to remove the free-text comments to avoid compromising the identity of some of the participants. The survey was pilot-tested among three regulatory science experts. No changes to the survey questions were required; however, some amendments were made to the front page of the survey and participant information sheet. The changes helped to clarify that all participants’ answers would be anonymous and their answers would not be identified. This study was approved by the ethical review committee at the University of Birmingham, UK (ERN_20-0268).

The participant information sheet [(Appendix 2) Electronic supplementary material] was provided to potential participants electronically prior to the survey completion. Survey participants were asked to give consent on the first page of the study; if consent was not given, they were not able to progress to the next part of the survey. The survey targeted national healthcare professionals, academics, patient advocates, health technology assessment agencies, ethicists and trade associations and international regulators, pharmaceutical companies and small or medium enterprises involved in regulatory science, either developing or applying regulation in a healthcare setting. Respondents were volunteers from a sample of eligible individuals recruited through stakeholder organisations and collaborators with relevant experience in regulatory science. Furthermore, stakeholders contacted were asked to refer further potential participants (snow-ball recruitment). Data were collected between April and May 2020. The survey was hosted by SmartSurvey™.

### Analysis

Descriptive quantitative analysis was undertaken for each respondent group. Frequency distributions were used to describe respondent characteristics and survey responses. SCR undertook direct content analysis of the free-text comments responses [13]. Survey data were managed using a qualitative data analysis software package (QSR NVivo 12). The analysis process started with reading the survey responses several times to increase familiarity with the data. This was followed by inductive coding process. The coding was reviewed by BT and any disagreements about the coding were discussed and resolved with a third reviewer (MJC, EM and AKD).

## Results

A total of 145 participants took part in the survey, and all responses were anonymised. Ninety-one percent (132/145) of the respondents were UK stakeholders while 7% (13/145) were international, mainly from the United States and Europe. Thirty-two questionnaires were partially completed. These were only included in the data analysis if they had responses completed to at least one of the key “Strategic areas for development” questions, as they provided essential information on the criteria areas for the development of UK regulatory science.

Table 1 outlines the characteristics of the participants. Stakeholders’ background included a wide range of clinical and methodological expertise, including pharmacovigilance and regulatory affairs. See Appendix 3 (Electronic supplementary material) for further details.

**Table 1.** Participants’ characteristics.

Stakeholder group	*n* (%), *N* = 145
Pharmaceutical company and large med-tech	31 (21)
Academic	26 (18)
Regulator	17 (12)
Healthcare professional	14 (10)
Patient representative	14 (9)
Small- or medium-size enterprise	9 (6)
Health Technology Assessment agency	5 (3)
Ethicists	3 (2)
Trade association	2 (1)
Other*	26 (18)

Years of experience in regulatory science	*n* (%)
Less than 1 year of experience	9 (6)
2 – 5 years	37 (25)
6 – 10 years	18 (12)
More than 10 years	81 (56)

^*^Other: regulatory consultancy, government body, clinical research organisation/practitioner, service provider to the pharma industry.

### Defining Regulatory Science in Healthcare

As noted, several international agencies such as the EMA and FDA and international networking groups like the Collaboration for the Advancement of Sustainable Medical Innovation (CASMI) and the PSJ have proposed different definitions of healthcare regulatory science. Stakeholders were asked to select the most accurate definition based on their understating of the discipline.

The most agreed definition among participants (50%) was the one provided by the EMA: “Regulatory Science can be described as a range of scientific disciplines that are applied to the quality, safety and efficacy assessment of medicinal products that inform regulatory decision-making throughout the lifecycle of a medicine. It encompasses basic and applied medicinal science and social sciences and contributes to the development of regulatory standards and tools” [14]. The next most supported was the CASMI definition (18%): “The application of the biological, medical, and sociological sciences to enhance the development and regulation of medicines and devices in order to meet the appropriate standards of quality, safety and efficacy” [1].

However, one participant (1%) commented that the most accurate definition of regulatory science should be a combination of the FDA and EMA definitions. One participant proposed combining the FDA and CASMI definitions, and another participant proposed combining the CASMI and PSJ ones. Two participants suggested modifying the EMA definition by adding the term ‘medical devices’ and one suggested substituting the term ‘throughout the life cycle’ for ‘any regulated product, service or system’. One participant suggested revising the FDA definition to broaden the term ‘FDA-regulated products’ to ‘medicines, devices and other health technologies’. One participant suggested adding the wording ‘that patients expect and deserve’ at the end of the CASMI definition. Lastly, three participants suggested their own definitions of regulatory science in healthcare.

### Areas of UK Healthcare Regulation that can be Enabled Through Regulation

Participants ranked the top six outcomes of future regulation which regulatory science needs to effectively enable (Table 2).

**Table 2.** Six most important outcomes of UK regulation and innovation which regulatory science needs to effectively enable.

Strategic outcomes	
1	Flexibility: the capability of regulations to adapt to novel products and target patient outcomes
2	Co-development: collaboration across sectors, e.g. patients, manufacturers, regulators and educators working together to develop appropriate training for novel product deployment
3	Responsiveness: the preparation of frameworks which enable timely innovation required by emerging events
4	Speed: the rate at which new products can reach the market
5	Reimbursement: developing effective tools to track and evaluate outcomes for “pay for performance” products
6	Education and professional development

Participants highlighted several challenges and opportunities that need to be addressed to drive the outcomes proposed in Table 2. The most common were as follows: cooperation between agencies (e.g. MHRA, EMA and FDA) 82% (119/145); dialogue/cooperation across stakeholders (e.g. academia, regulatory agencies and patients/patient advocates) 76% (110); development of a framework to make regulatory decisions about risks and benefits of products that increasingly involve new technology (e.g. digitally based products/AI and product development, production processes and novel supply chains) and target patient outcomes 70% (102); data sharing 63% (92); and technological and scientific challenges—genomics and increased personalisation/specialisation of products 50% (73). See Appendix 4 for further challenges and opportunities.

### Strategic Areas for Development

Table 3 presents the top three priorities that participants considered the most important for the development of UK regulatory science according to the following strategic areas identified in the MHRA corporate plan: (a) patient-centred drug development; (b) enhancing innovation; (c) proactive, robust surveillance; and (d) organisational excellence/efficiency.

**Table 3.** Top three priorities per strategic areas of development.

Patient-centred drug development	*n* (%), *N* = 135
Ensure that patients’ experiences, perspectives, needs, and priorities are captured and meaningfully incorporated into drug development and evaluation	104 (77%)
Enhance understanding and appropriate use of methods to capture information on patient preferences and the potential acceptability of trade-offs between treatment benefit and risk outcomes	84 (62%)
Reinforce patient relevance in evidence generation, increased patient involvement in regulatory activities, including patient representatives as additional experts in Scientific Advisory Groups, as well as patient contribution to scientific advice and protocol assistance	81 (60%)

Enhancing innovation	*n* (%), *N* = 131
Explore innovative ways of using real-world data to assess clinical effectiveness in routine clinical settings	86 (66%)
Explore developing standards for new areas, e.g. digital health, artificial intelligence, including machine learning	84 (64%)
Explore developing more agile regulatory approvals processes for novel and generic products	79 (60%)

Proactive and robust surveillance	*n* (%), *N* = 125
Optimise signal and risk assessment functions to respond to risks in real time	75 (60%)
Enhance information sharing	70 (56%)
Develop systems and processes for integrated medicines and devices surveillance	68 (54%)

Organisational excellence/efficiency	*n* (%), *N* = 124
Explore opportunities to develop collaborations and information sharing with key global regulators, international partnerships with WHO (World Health Organisation) and other key players	92 (74%)
Invest in scientific capabilities to meet emerging needs. Invest in staff’s specialist skill sets, and in facilities to deliver state of the art regulation and services	87 (70%)
Identify future capability needs and ensure the right skill mix is available to support innovation and deliver priority programmes and core functions	83 (67%)

### Existing Cross-Sector Training and Workforce Development Needs

The participants reported the need for life-long learning to keep pace with the breath and dynamism of regulatory science. This learning and development should be responsive to the constant advancement of innovative technologies and products. Fifty-five percent (68/124) of respondents were unaware of any training courses or training schemes in regulatory science. Training opportunities in UK (45%, 56/124), in the United States (26%, 32/124), and in other European countries (19%, 24/124) were marked as most commonly known to the responders. The most frequently attended courses by the respondents were these delivered by The Organisation for Professionals in Regulatory Affairs (TOPRA), the MHRA, FDA, EMA, Pharmaceutical Information and Pharmacovigilance Association (PIPA), Drug Information Association (DIA) and King’s College London University. Please see Appendix 4 (Electronic supplementary material) to see other courses mentioned by the participants.

The survey results showed that 67% (83/124) of participants had not attended training courses. The reasons listed included the following: (a) lack of awareness of the courses, (b) lack of funding for attendance, (c) courses not meeting the stakeholders’ needs or not being relevant to them, (d) time constraints and (e) courses not being freely available for patients/patient advocates. Participants confirmed that training is essential to meet the current (79%, 94/119) and future (85%, 94/119) needs of regulation. They suggested that regulatory science education and training should be delivered at continuing professional development (CPD) courses (87%, 104/119), postgraduate taught Master of Science (MSc) degree (62%, 74/119), postgraduate research Master of Research (MRes) or PhD degree (52%, 62/119), cross-sector fellowships (48%, 57/119) and academic fellowships (45%, 53/119).

Stakeholders expressed that additional training formats such as online training, short intensive courses, short courses at conferences and big meetings, training for patients/patient advocates and free webinars would be beneficial to advance regulatory science. The following topics were proposed: biologics and advanced therapies, ethics and governance, new medical device regulations, omics and its use in clinical and regulatory science and understanding AI and machine-learning processes.

### Infrastructure Required

#### Access to Expertise

In terms of regulatory science infrastructure, the majority of participants (85%, 101/119) expressed that they know how to access expertise when working in a highly regulated area. Additionally, 76% (91/119) of the participants had knowledge of the MHRA Innovation Office although only 24% (28/119) had accessed this support.

#### Successful Cross-Sector Collaborations and Barriers in Creating Collaborations

Survey respondents provided several examples of national and international collaborations for the development of health products across disciplines. These included but were not limited to: (i) international joint efforts to tackle COVID-19 by developing vaccines, ventilators and conducting further relevant research, (ii) development of a machine-learning algorithm for eye disease diagnosis, (iii) AI for breast cancer screening and (iv) predicting patients at risk of sudden cardiac death after myocardial infarctions. Table 4 presents different elements that made these and other collaborations successful and examples of challenges that survey responders have encountered such as legal, structural and ethical barriers while developing cross-sector collaborations.

**Table 4.** Key considerations and barriers in collaboration on health products development.

Considerations	Barriers
● Strong and cross-sector collaborations (e.g. academia, patient-representative bodies/charities, regulators and industry) ● Early engagement and communication with stakeholders ● Patient and public involvement ● Availability of funding ● Recruitment and availability of appropriate expertise ● Stakeholders’ aligned strategies and common purpose ● Flexibility in considering different stakeholders’ perspectives ● Transparency ● Perspectives of end users well captured and integrated into the research design ● Training available related to the research being conducted ● Adapting to changing regulatory environment	Legal barriers ● Intellectual property (IP) concerns and ownership ● Data sharing, access agreements and confidentiality ● Difficulty agreeing terms for contracts between academia and industry (e.g. data sharing) Structural barriers ● Differences between regulatory requirements between Europe, the UK and US ● Differences in regulatory requirements for CT products and medical devices ● Uncertain regulatory guidance around AI and ML algorithms ● Multiple levels of clearance/review in regulatory agencies ● Lack of capacity from MHRA regulatory, specifically in the medical devices area ● Conservatism or ‘old school’ thought process prevalent among companies and agencies ● Insufficient funding to support a full research project Other barriers ● Lack of information sharing between MHRA and NICE ● Lack of trust between health systems (e.g. NHS) and pharmaceuticals ● Language barriers encountered in contracts which delays the research process ● Potential conflict of interest as an impediment/prevention from collaboration taking place (e.g. inability of third-party independent organisations to accept money from industry) ● Excessive time needed to reach collaborative agreement ● Excessive workload as barrier to CPD and career development ● Cultural differences

### Academic Leadership

The need for academic leadership in the UK in developing cutting-edge technologies to evaluate safety, efficacy, quality and performance of new health products and technologies was expressed by 80% of the responders (92/115). According to survey respondents, the main areas that would benefit from academic leadership, currently and in future, are innovative trial design (68%, 78/114—mainly supported by academics (57%, 15/26) and regulators (88%, 15/17)), real-world evidence (67%, 76/114—mainly supported by academics (57%, 15/26)), patient-centred technology development (64%, 73/114—mainly supported by academics (46%, 12/26), healthcare professionals (85%, 12/14) and patient representatives (85%, 12/14), and AI in healthcare (62%, 71/114—mainly supported by academics (65%, 17/26 and regulators (64%, 11/17)). Furthermore, participants proposed additional areas such as data ethics, advanced therapy medicinal products, genomics and regulation of clinical trials.

In addition, some participants suggested that academia can play a leadership role in new methods development, particularly around the regulation and ongoing evaluation of new technologies where the existing regulatory framework is insufficient, such as AI and digital health technologies. Survey participants reinforced a need for academia to provide regulatory courses for workforce development, particularly to benefit clinicians, industry and regulators. Eight participants noted the need for academic leadership in collaboration with industry, health professionals, regulators and patient advocates to develop effective and appropriate regulation of innovative healthcare products.

## Discussion

This survey aimed to assess stakeholder views on the definition of regulatory science and explore critical challenges and opportunities in regulatory science for UK healthcare. Several key messages emerged. Firstly, there was a lack of agreement upon the existing and proposed definitions of regulatory science in healthcare although a significant buy-in to the existing EU-level definition was seen. The current definitions address specific stakeholder needs, for example the EMA definition is focussed, unsurprisingly, on medicines, whilst the patient perspective is not often mentioned. Thus, there is a need for further discussion to seek agreement on the most appropriate definition of regulatory science in healthcare in the UK in order to underpin a coherent future strategy, incorporating views from a range of stakeholders. In addition, it is necessary to discuss the different subdisciplines embraced within the definition of regulatory science, proposed by the EMA and CASMI, referring to medical, biological and sociological sciences.

The most important feature of future UK regulation identified by survey participants was flexibility (Table 2)—the capability of regulations to adapt to novel products and target patient outcomes facilitated through cooperation between relevant agencies, which is essential to enable some element of regulatory science future-proofing [9]. This mirrors international trends supporting increased globalised development of health products through collaborative working across international regulatory agencies. Since 2003, when a memorandum of understanding and confidentiality agreement was signed between the FDA and EMA, these two and subsequently other regulatory agencies have been meeting to discuss experiences and developments of regulatory science [15]. Over the years, formal clusters of such information exchange, many underpinned by legal agreements, have been created to cover many therapeutic areas or types of products. The regulatory authorities remain in close, nearly daily, dialogue [15]. Up until the UK’s departure from the EU, the MHRA has been part of these collaborations representing the UK as part of the EMA network. It is vital that the UK’s participation, profile and leadership of such international discussions are not only preserved but further enhanced where possible if the UK expects to remain a globally competitive marketplace and supporter of innovation in healthcare.

Other important features to facilitate advances in regulatory science were “Broader cooperation and dialogue between stakeholders such as academia, industry, regulatory agencies and patients”, followed by “Development of a framework of regulatory decision-making on weighing risks and benefits of products involving new technologies” and “Data sharing”. These features again emphasise the value of collaborative efforts in managing both UK and global healthcare innovation, and the importance of relevant regulations to keep pace not only with the emerging technologies but also with the development of appropriate methods of their assessment, which is a whole new science in its own right. In particular, this highlights the need to create systems which can appropriately provide assurance of adaptive algorithms’ performance, which will change over time and across populations, learning from the data to which they are exposed. Regulation of such systems cannot simply be more of the same, but is likely to benefit from regulatory innovation that is based on a deep understanding of the technology [16].

The existing UK industry sectors have a “very real and concerning lack of readiness” [17] for new regulations, even while flexibility and innovation in future regulations have been prioritised in key recent strategic recommendations including the UK R&D Roadmap [18] and the Life Sciences Recovery Roadmap supported by the UK’s industry trade associations [19]. The responses to the survey clearly highlight the importance of cross-stakeholder working to overcome these challenges in a timely and effective way.

Survey participants recognised the importance of patient-centred drug development, championed by both the MHRA and EMA. This recognition reinforces the consistent message for greater involvement of patient advocates in regulatory activities and therapeutic development [20, 21]. Increased partnership, collaboration and engagement of patients and the public have the potential to ensure that patients receive timely access to innovative products and that health care providers have the information needed to inform healthcare choices. This can be achieved by improving the way regulators share and communicate safety messages to clinicians and patients, optimising licensing pathways and exploring opportunities to develop more agile, transparent and joined up approval procedures [6, 20]. In addition, methodological issues surrounding the subject of capturing patients’ preferences and acceptability of trade-offs were raised. Improvement of methods is necessary to enhance the understanding of their appropriate use and interpretation. Together, promoting patient-centred drug development and enhancing methodological issues create a powerful, cross-stakeholder call to action for the UK to develop effective mechanisms for working closely with patients and the public to build public trust, robust discussion and input, and shared understanding of the importance of effective regulation.

In the area of enhancing innovation, the need for exploring ways of using real-world data to assess clinical effectiveness in routine clinical setting was valued most highly by survey participants, according to the MHRA corporate plan’s strategic areas of development. Other prioritised actions were the development of standards for new technologies and the need for a more agile approval process. Optimising signal and risk assessment functions to respond to risks in real time was considered most important within the proactive and robust surveillance strategic area of development. All of this speaks to the need for flexibility, efficiency and an acceleration of regulation which responds to emerging data for novel technologies rather than relying on existing frameworks, regulatory or otherwise, which may in some instances be outdated. Further examination of the potential to couple implementation, evaluation and regulation in the UK’s future strategy could be a highly valuable focus for regulatory science.

Stakeholders recognised that it is essential to develop collaboration and share information with key international regulators and partner with key players such as WHO, when thinking of the development of regulatory sciences in organisational excellence and efficiency. In addition, they highlighted the need of investing in workforce skills and facilities to deliver novel regulatory services. This echoes previous points about the value of considering the UK’s future strategy in a global context and particularly the development of a workforce capable of understanding international comparators and collaborators to achieve the best outcomes. Regulatory experts should work in close partnership with relevant regulatory service providers, learning from one another to maximise relevance and responsiveness.

Regulatory science training is essential due to the rapid emergence of innovative technology and products; however, the survey identified that 67% of the participants have not attended any regulatory science training, most commonly due to lack of awareness of its existence. Potential reasons for stakeholders not looking into training are lack of employer’s funding, stakeholders’ lack of time to commit to the course length and the courses not being relevant to the stakeholders’ career needs.

Stakeholders expressed that regulatory training should be mainly delivered through CPD courses. However, the need for formal education such as MSc or PhD programmes was also suggested. Topics covering real-world evidence, biologics and advanced therapies, AI and ethics and governance were especially in demand. This reflects the need for both regulators and innovators to understand emerging technologies—and the barriers and enablers which will allow their reach through to patients—through a consistent, shared training framework. Participants were supportive of this being led by academia; however, the benefits highlighted elsewhere of cross-sector collaboration, particularly with patients and the public, would certainly be critical to ensuring this is as relevant and responsive as possible. A list of existing national and international educational programmes is available at the Advancing Regulatory Science and Innovation in Healthcare report by Birmingham Health Partners [22].

For UK regulatory science to retain a competitive and collaborative position within the global market post-Brexit, participants reinforced the need for strong, cross-sector collaboration to develop critical infrastructure involving academia, patient-representative bodies, charities, regulators and industry. However, multiple barriers make such collaborations difficult. Among them, most commonly identified were various legal restrictions especially those around intellectual property, data sharing and access not only with international partners but even internally within the UK. In addition, the UK must continue to probe these stumbling blocks and look to adopt or create good practice which can be shared globally to better enable the collaborations which are clearly vital to future regulatory innovation.

Academic leadership in development of regulatory science, which would allow regulators to more effectively keep pace with scientific development, was supported by the majority of participants. Importantly, academia was not perceived as the sole driving force but rather as a partner in collaboration with other stakeholders. The areas of particular need for academic input were innovative trial designs, real-world evidence, patient-centred technology development and AI. Partnership between academia and regulators is essential to enable and leverage research and innovation in regulatory science [6]. UK universities are internationally recognised for their research in healthcare, law, ethics, social policy, health economics, engineering, business, biological sciences and chemistry, among other multidisciplinary programmes. Expertise from each of these should be contributing to regulatory innovation. However, only a small number of universities teach aspects of regulatory science, and none of these cover a fraction of the breadth of disciplines outlined above [22]. The UK has the potential to develop a regulatory science fellowship programme similar to the one provided by the Oak Ridge Institute for Science and Education (ORISE) Research Participation Program at the FDA [23]. This educational and training programme aims at providing college students, recent graduates and university faculty opportunities to access unique research and training opportunities, state of the art facilities and equipment and staff in order to more effectively implement novel technologies within healthcare.

This survey presented some limitations. The sampling method was not random but based on pre-selected stakeholder representatives allowing for the snow-balling approach, which was considered more appropriate for such a highly technical subject. The authors acknowledge that their interest in this area may have influenced the framing and approach to the survey; please refer to the ‘competing interest’ section below. The survey did not assess the need for continued maintenance of standards in regulatory science for conventional evaluation methodologies for drugs and devices, such as various forms of trial and comparative study designs. One patient representative expressed that the language used throughout the survey was not friendly enough for participants and there were some irrelevant questions for them. However, the rest of the patient partners completed the survey without complications or raising any other concerns. In addition, there was some missing data within the dataset possibly affecting the robustness of the analysis.

This study evaluated the priorities for the UK’s regulatory science agenda post-Brexit and through the lens of the UK’s continuing response to COVID-19, particularly in the context of global opportunities. The authors find that trust, collaboration, leadership and training are vital for the UK’s current and future international position, both as a competitive marketplace for innovation but also in supporting the voice of patients and the public as the UK accelerates safe and effective advances in healthcare; particular attention should be given to novel and innovative developments. This is a unique, challenging time for the UK—with reason to be optimistic if it can meet the challenges and opportunities outlined here with an open mind, clear strategy and commitment to partnership.

## Supplementary Information

Below is the link to the electronic supplementary material.

Electronic supplementary material 1 (PDF 100 kb)

Electronic supplementary material 2 (PDF 77 kb)

Electronic supplementary material 3 (DOCX 26 kb)

Electronic supplementary material 4 (PDF 591 kb)
