# Supplementary material for: Advancing UK Regulatory Science Strategy in the Context of Global Regulation: a Stakeholder Survey
Source: Ther Innov Regul Sci. 2021 Feb 16;55(4):646–55. doi: 10.1007/s43441-021-00263-2 (PMC7885762; doi:10.1007/s43441-021-00263-2)
Supplement: Supplementary file 1 — Electronic supplementary material 1 (PDF 100 kb) [file 43441_2021_263_MOESM1_ESM.pdf]

1. Challenges and strategic areas of regulatory science in the UK

Recent years have seen the development of innovative healthcare products including genome- and cell-based therapies, mobile health products, artificial intelligence and implantable devices. This survey seeks to understand how regulatory science in the UK and beyond can remain at the cutting edge of increasingly-complex innovations so that they can be translated safely and swiftly into effective, high-quality therapies (Hines P. et al, 2019).

Prior to completing the survey please read the **participant information sheet (https://drive.google.com/file/d/1byBV0Il0-JQPhafMqmY8ip4dkVUC6BvF/view?usp=sharing)** sent to you in the invitation email – in summary:

> Your participation in this survey is voluntary. We anticipate it will take approximately **15 minutes** to complete. You can **save your progress** and access the survey to complete at a later date if you wish. An email will be sent to you with the relevant link. All information that is collected about you during the course of the research will be **anonymised at individual level**, kept **confidential** and **stored securely**. We aim to publish the results in relevant scientific journals. You **will not be identified** in any report or publication. You may **withdraw** without giving a reason up to **7 working days** after submission of the study. If you wish to withdraw, please contact Samantha Cruz Rivera (s.rivera@bham.ac.uk) quoting the **unique ID** generated on the first page of the survey.
> If you have concerns about any aspect of this study, please contact Samantha Cruz Rivera (s.rivera@bham.ac.uk) or the principal investigator Professor Melanie Calvert (+44 121 414 8595 or m.calvert@bham.ac.uk), who will do their best to answer your questions.

By progressing to the next page you confirm that you have read the information sheet and consent to take part of the study.

Thank you for taking the time to participate in this survey.

Yours sincerely,
Melanie Calvert, PhD

Professor of Outcomes Methodology
NIHR Senior Investigator
Director Centre for Patient Reported Outcomes Research
Institute of Applied Health Research
University of Birmingham Edgbaston Birmingham

2. Demographics

1. In order to keep your answers anonymised please generate your own unique ID. This should be formed by two letters and four numbers of your choice (e.g. SC1909) *

2. Please select the main stakeholder group you belong to: *

- ☐ Healthcare professional
- ☐ Academic
- ☐ Patient representative
- ☐ Health technology assessment agency
- ☐ Regulator
- ☐ Pharmaceutical company
- ☐ Large med-tech
- ☐ Small or Medium Size Enterprise
- ☐ Ethicist
- ☐ Trade association (please state)
- ☐ Other (please specify):

Please specify your disease or methodological area of expertise: *

3. Approximately how many years experience do you have in the development or application of regulation *

- ☐ 1 year or less
- ☐ 2 - 5 years
- ☐ 6 - 10 years
- ☐ More than 10 years

3. Defining regulatory science in healthcare

4. Below are presented different definitions of regulatory science. Which one do you think provides the most accurate definition based on your understanding of the discipline? *

☐ "Regulatory Science is the science of developing new tools, standards, and approaches to assess the safety, efficacy, quality, and performance of all FDA-regulated products." (FDA, 2010)

- "Regulatory Science can be described as a range of scientific disciplines that are applied to the quality, safety and efficacy assessment of medicinal products that inform regulatory decision-making throughout the lifecycle of a medicine. It encompasses basic and applied medicinal science and social sciences and contributes to the development of regulatory standards and tools" (EMA, 2018)
- "The acquisition and analysis of data sufficient to inform decision making pertinent to the approval of safe and effective therapeutics, devices and cosmetics and ensuring the safety and nutritional value of the food supply." (FitzGerald, 2010)
- "The science of developing methods to quantitatively and/or qualitatively analyze and understand the causal relations and mechanisms of the substances and the phenomena around us, and measuring their positive and negative effects. Their efficacy and safety are appropriately predicted, evaluated and judged using the methods developed and the results obtained in the [regulatory science], contributing to public health." (The Pharmaceutical Society of Japan, 2016)
- "The application of the biological, medical and sociological sciences to enhance the development and regulation of medicines and devices in order to meet the appropriate standards of quality, safety and efficacy." (CASMI, 2013)
- Other (please specify):

4. Current challenges and opportunities in regulatory science

5. What are the most important features of future regulation for UK healthcare innovation, which regulatory sciences needs to more effectively enable. Select all that apply *

- Data sharing
- Open access
- Costs associated to data sharing (e.g. the costs of anonymising data)
- Cooperation between agencies (e.g. MHRA, EMA and FDA)
- Dialogue/cooperation across stakeholders e.g. between academia – industry (including biopharma and med tech) – regulatory agencies – patients
- Remaining aligned with EU following the UK’s exit from the European Union
- Faster approval of medical products
- Development of a framework to make regulatory decisions about risks and benefits of products that increasingly involve new technology (e.g. - digitally-based products/AI and product development, production processes and novel supply chains) and target patient outcomes.

☐ Technological and scientific challenges -genomics and increased personalisation/specialisation of products

☐ Developing effective tools to track and evaluate outcomes for reimbursement

☐ Professional development (i.e. workshops or courses to supplement knowledge base)

☐ Workforce retention rates

☐ Other (please specify):

5. Current challenges and opportunities in regulatory science

6. Please rank (drag and drop the question boxes) the most important features of future regulation for UK healthcare innovation, which regulatory sciences needs to more effectively enable: *

Co-development: collaboration across sectors, e.g. patients, manufacturers, regulators and educators working together to develop appropriate training for novel product deployment

Responsiveness: the preparation of frameworks which enable timely innovation required by emerging events

Speed: the rate at which new products can reach the market

Flexibility: the capability of regulations to adapt to novel products and target patient outcomes

Reimbursement: developing effective tools to track and evaluate outcomes for "pay for performance" products

Education and professional development

6. Strategic areas for development

The Medicines and Healthcare Products Regulatory Agency’s Corporate Plan 2018-2023 identifies a number of actions, activities and projects related to Regulatory Science.

7. Thinking about your stakeholder group, which of the actions below are most important for the development of UK regulatory science. Select all that apply Patient Centered Drug Development *

☐ Ensure that patients’ experiences, perspectives, needs, and priorities are captured and meaningfully incorporated into drug development and evaluation.

- [ ] Reinforce patient relevance in evidence generation, increased patient involvement in regulatory activities, including patient representatives as additional experts in Scientific Advisory Groups, as well as patient contribution to scientific advice and protocol assistance.
- [ ] Enhance understanding and appropriate use of methods to capture information on patient preferences and the potential acceptability of tradeoffs between treatment benefit and risk outcomes.
- [ ] Increase use and transparency of patient input as evidence in regulatory science decision-making.
- [ ] Expand patient centered outcomes research by compiling datasets converted to standardized format across critical classes of drugs that are entered into the clinical trials repository.
- [ ] Identification and use of approaches and best practices to facilitate patient enrollment and minimizing the burden of patient participation in clinical trials.
- [ ] Other (please specify):

7. Strategic areas for development

8. Thinking about your stakeholder group, which of the actions below are most important for the development of UK regulatory science. Select all that apply Enhancing innovation *

- [ ] Develop processes and maximise expertise in dealing with special/small populations, pragmatic trials and real world data
- [ ] Underpin cost effective research using real world data
- [ ] Develop/implement innovative methodologies and deliver comprehensive research to underpin standardization, control and innovation of biological medicines
- [ ] Explore further opportunities for reclassification of medicines
- [ ] Explore new technology projects on digital products, artificial intelligence/machine learning,
- [ ] Support the development of Advanced Therapies Medicinal Products (ATMPs) and novel biologicals by providing useful standards and all-stage expert scientific advice
- [ ] Develop new standards e.g. for biologicals including biosimilars, advanced therapies
- [ ] Support innovative strategies to reduce the use of antibiotics, e.g. implementation of novel vaccines and therapies
- [ ] Engage with the clinical diagnostics community to enhance reliability of disease diagnoses and commutability of results

- [ ] Explore innovative ways of using real world data to assess clinical effectiveness in routine clinical settings
- [ ] Explore developing more agile regulatory approvals processes for novel and generic products
- [ ] Explore developing standards for new areas e.g. digital health, artificial intelligence, machine learning
- [ ] Explore supporting opportunities in vaccines, combination products, software algorithms, remote site reporting and additives
- [ ] Other (please specify):

8. Strategic areas for development

9. Thinking about your stakeholder group, which of the actions below are most important for the development of UK regulatory science. Select all that apply Proactive, robust surveillance

- [ ] Develop systems and processes for integrated medicines and devices surveillance
- [ ] Optimise signal and and risk assessment functions to respond to risks in real time
- [ ] Develop professional expertise and systems to improve market surveillance of medical devices using the new regulations and its new data sets
- [ ] Develop and expand use of medical device electronic data standards with partners
- [ ] Encourage reporting of adverse incidents from patients and health care professionals
- [ ] Systematically evaluate effectiveness of risk minimisation and impact
- [ ] Work closely with key source countries to assure safe production and supply
- [ ] Enhance information sharing
- [ ] Enhance the role of pharmacovigilance in enabling the introduction of innovative medicines

- [ ] Other (please specify):

9. Strategic areas for development

10. Thinking about your stakeholder group, which of the actions below are most important for the development of UK regulatory science. Select all that apply Organisational excellence/efficiency *

- [ ] Identify future capability needs and ensure the right skill mix is available to support innovation and deliver priority programmes and core functions
- [ ] Identify opportunities to recruit, retain and develop staff; including new training opportunities
- [ ] Invest in scientific capabilities to meet emerging needs. Invest in staffs specialist skill sets, and in facilities to deliver state of the art regulation and services.
- [ ] Focus on identifying and developing talent and on the importance of leadership, in the context of organisational development in an environment of scientific advance and environmental change
- [ ] Explore new methodologies for detecting data integrity issues associated with regulatory studies
- [ ] Explore opportunities to develop collaborations and information sharing with key global regulators, international partnerships with WHO and other key players
- [ ] Other (please specify):

10. Current training and future needs

Regulatory science encompasses a wide range of subjects, including not only disciplines associated with regulation, such as statistics, engineering and clinical research, but also disciplines outside the biomedical sciences such as informatics, economics, risk communication, and sociology.

Please state whether you agree with the following statements:

11. Due to its breadth and dynamism, regulatory sciences require life-long learning* and development. *Life-long learning: Voluntary and self-motivated education or learning, beyond formal education, to improve personal or professional development. *

- [ ] Yes

- [ ] No

12. This learning and development needs to be responsive to the development of innovative technologies and products. *

- [ ] Yes
- [ ] No

11. Current training and future needs

13. Are you aware of any regulatory science training courses or training schemes (programmes) in: Select all that apply *

- [ ] United Kingdom
- [ ] United States
- [ ] Any of the countries in the European Union
- [ ] Any other country
- [ ] I'm not aware of any of these training courses
- [ ] Other (please specify):

12. Current training and future needs

14. Have you attended any of these training courses before? *

- [ ] Yes
- [ ] No

13. Current training and future needs

15. Please state the reasons why you haven't attended to any of these courses: *

14. Current training and future needs

16. Please state which courses you have attended and why: *

15. Current training and future needs

17. What kind of additional/novel training formats and topics do you think regulatory science would benefit from? *

18. Would these training be useful for current or future regulatory science needs? *

- [ ] For current priorities
- [ ] For future priorities

16. Current training and future needs

19. At what level of education and training do you think regulatory science should be focused? Select all that apply *

- [ ] Undergraduate

- [ ] Postgraduate - Taught MSc
- [ ] Postgraduate - Research MRes or PhD
- [ ] Academic fellowships
- [ ] Cross-sector fellowships
- [ ] Continuing Professional Development (short-course)

17. Infrastructure required

20. When working in a highly regulated area, do you know how to access expertise? *

- [ ] Yes
- [ ] No

18. Infrastructure required

21. If you are to search for such expertise, would your starting point be the UK or international market? *

- [ ] UK
- [ ] International market

19. Infrastructure required

22. Are you aware of the MHRA Innovation Office? *

- [ ] Yes
- [ ] No

23. Have you accessed them? *

- [ ] Yes
- [ ] No

20. Infrastructure required

24. From your experience, can you give examples of successful collaborations on health products development across sectors in the UK or internationally? *

25. What elements made these a success? *

21. Infrastructure required

26. What legal, structural and other barriers have you experienced in creating collaborations? *

22. Infrastructure required

27. Do you see the need for academic leadership in the UK in developing cutting-edge technologies to evaluate safety, efficacy, quality, and performance of developed new health products and technologies? *

- [ ] Yes
- [ ] No

Please provide further details:

23. Infrastructure required

28. Would you consider any of these area as particularly benefiting from academic leadership? Select all that apply *

- ☐ Patient-centred technology development (including: patient reported outcomes, patient and public involvement and "rise of the small population")
- ☐ AI in healthcare
- ☐ Real World Evidence
- ☐ Accelerated access to medicines
- ☐ Innovative trial design
- ☐ Do you consider any other areas as particularly requiring academic input or leadership? Please specify
- ☐ Other (please specify):

24. Infrastructure required

29. The US offers a range of fellowships in regulatory sciences developed in cooperation between academia, FDA and industry. Do you see such a need in the UK market? *

- ☐ Yes
- ☐ No

25. Additional information

30. Do you have any other comments you would like to add?

26. Additional information

31. Please provide your email if you would like to be receive future publications arising from the data collected:

27. Additional information

32. Would you like to be acknowledged in future publications? *

- [ ] Yes
- [ ] No

28. Additional information

33. Please provide your name and affiliation in the way we should acknowledge you in the publication:
