# Supplementary material for: Advancing UK Regulatory Science Strategy in the Context of Global Regulation: a Stakeholder Survey
Source: Ther Innov Regul Sci. 2021 Feb 16;55(4):646–55. doi: 10.1007/s43441-021-00263-2 (PMC7885762; doi:10.1007/s43441-021-00263-2)
Supplement: Supplementary file 2 — Electronic supplementary material 2 (PDF 77 kb) [file 43441_2021_263_MOESM2_ESM.pdf]

Participant Information Sheet (online survey)

Challenges and strategic areas for the development of the UK regulatory science

Prof Melanie Calvert, University of Birmingham
Dr Eliot Marston, University of Birmingham
Dr Samantha Cruz Rivera, University of Birmingham
Dr Barabara Torlinska, University of Birmingham

WHAT IS THE PURPOSE OF THIS STUDY?

Regulatory science and practice need to be responsive to emerging changes in technology, clinical practice and societal/public needs, many of which are rapidly moving with an explosion of advances in fields such as digital devices, personalised medicine, health data and AI, as well as novel trial design and patient centricity in drug development. To meet these challenges and grow the UK's global leadership position in industrial, academic and regulatory innovation, enhanced cross-sector partnership working will be critical.

Your professional opinion is needed to identify the current challenges in regulatory science in healthcare. We wish to identify strategic areas for the development of regulatory standards, establish current and future training needs and identify the infrastructure required to support this. Taking part in this online survey should take no longer than 20 minutes to complete.

Who are we?

This project is led by and funded by the University of Birmingham.

Why are we approaching you?

We are inviting people who have experience in developing or using regulatory guidance in their work, this can include those responsible for regulation and those who adhere to regulation in their research.

WHAT WILL HAPPEN TO ME IF I TAKE PART?

Online survey

If you are interested in taking part in this research project, please follow the link included in this email and complete the survey. The survey can be completed at individual or at organisational level. The survey should take no longer than 15-20 minutes to complete. The questions contained within the survey are focused on current challenges in regulatory science, strategic areas for development, current training and future needs and; infrastructure required to support this. You will be asked to give consent on the first page of the study; if you do not give it you will not be able to progress to the next part of the survey.

WHO IS ORGANISING AND FUNDING THE RESEARCH?

The study is being funded by the University of Birmingham and supervision of the study is being carried out by the University of Birmingham, UK.

WHO HAS REVIEWED THE STUDY?

The study **(ERN_20-0268)** approved on 04/03/2020 by the University of Birmingham Ethics Review Committee.

DATA PROTECTION

In order to carry out the project described above, we will need to collect information about you, and some of this information will be your personal data. Under data protection law, we have to provide you with very specific information about what we do with your data and about your rights.

The University of Birmingham's web page 'Data Protection - How the University Uses Your Data' sets out much of this information, including how to ask any questions you may have about how your personal data is used, exercise any of your rights or complain about the way your data is being handled. The rest of the key information you need to know about how we used your personal data is set out below.

Who is the Data Controller?
The University of Birmingham, Edgbaston, Birmingham B15 2TT is the data controller for the personal data that we process in relation to you.

What data are we processing and for what purpose will we use it?
All data collected from you during the consultations will be kept confidential by the researchers. Only researchers at the University of Birmingham involved in the study will have access to the online survey data.

The project team may wish to attribute quotes or key information to individuals or their host organisation in arising publications. In this instance, we will contact you to seek explicit consent by email prior to any publication. You will have the opportunity to review the text, to seek any internal approvals, to request addition of disclaimers and be acknowledged as a contributor in the publication.

What is our legal basis for processing your data?
The legal justification we have under data protection law for processing your personal data is that it is necessary to do so for our project, which is a task we carry out in the public interest.

How long will my personal data be kept?
Your data will be retained for 10 years after the publication of the project outcomes after which time it will be securely destroyed.

What will happen to the results of the project?
We aim to publish the results in relevant scientific journals. You will not be identified in any report or publication. We can keep you informed on any publications arising from this study if you wish. Simply complete the relevant section in the online survey asking for your contact details and we will keep you up-to-date when the results become available.

If you complete the survey at organisational level, the data will not be anonymised as you will be asked to state on behalf of what organisation you are responding. However, the data provided will not be attributed to the organisation or any other individual. In addition, we would like to acknowledge your contribution as organisation or individual in arising publications. If you agree with this, complete the relevant section in the online survey asking for your name and affiliation. This information will not be used to attribute any data collected.

Can I change my mind about taking part?
Yes. Participation is voluntary and you may withdraw from the study at any time without giving a reason. If you choose to withdraw after 7 working days after the survey has been submitted, data collected will be retained and used in the project.

Can I withdraw my data?
Participants and organisations will be able to generate their own unique ID using two letters and four number of their choice (e.g. SC1909) at the beginning of the survey. You can withdraw your information without giving a reason up to 7 working days after the survey has been submitted using this ID. Participants who wish to withdraw should email the Samantha Cruz Rivera (s.rivera@bham.ac.uk) including their unique ID and expressing their wish to withdraw. Once the withdrawal email has been received all the data and information provided by the participant will be deleted.

Will I receive any compensation for participating?
Participation is voluntary.

Who can I contact should I want to ask questions?
The project team will be happy to answer any questions you may have. Please see contact details below:

Primary point of contact: Dr Samantha Cruz-Rivera, Research Fellow, CPROR
Email: s.rivera@bham.ac.uk | Phone: 0121 414 3872

Other contact details:
Principal Investigator: Prof Melanie Calvert, Director, CPROR
Email: m.calvert@bham.ac.uk | Phone: 0121 414 7866

In the event of a complaint please contact:
University of Birmingham Research Ethics Officer: Susan Cottam
Email: s.l.cottam@bham.ac.uk | Phone: 0121 414 8825

By following the survey link and completing the survey you confirm that you have read and understood this information, and you will be asked to consent to take part in this study on the first page of the survey. If you do not give consent, you will not be able to progress to the next part of the survey.

If you are interested in completing the **survey at organisational level**, please email us back to provide you with the relevant link.

Individual survey: https://www.smartsurvey.co.uk/s/RegulatoryScienceIND/

Thank you for taking the time to consider participating in our project
