# Supplementary material for: Advancing UK Regulatory Science Strategy in the Context of Global Regulation: a Stakeholder Survey
Source: Ther Innov Regul Sci. 2021 Feb 16;55(4):646–55. doi: 10.1007/s43441-021-00263-2 (PMC7885762; doi:10.1007/s43441-021-00263-2)
Supplement: Supplementary file 3 — Electronic supplementary material 3 (DOCX 26 kb) [file 43441_2021_263_MOESM3_ESM.docx]

**Appendix 3**

**Stakeholders’ area of expertise***

| **Disease area** | **n (%)** |
| --- | --- |
| Oncology | 18 (12%) |
| Ophthalmology | 5 (4%) |
| Gastroenterology | 4 (3%) |
| Hepatology | 2 (1%) |
| Immunology | 2 (1%) |
| Neurology | 2 (1%) |
| Rare diseases | 2 (1%) |
| Cardiovascular | 1 (0.6%) |
| Fibrosis scarring | 1 (0.6%) |
| Haemostasis | 1 (0.6%) |
| Respiratory | 1 (0.6%) |
| Odontology | 1 (0.6%) |
| **Methodological area** | **n (%)** |
| Medical devices (includes digital medical devices) | 14 (10%) |
| Pharmacovigilance and pharmacoepidemiology | 12 (8%) |
| Regulatory affairs | 9 (6%) |
| Clinical Trials | 9 (6%) |
| Advanced and Cell and Gene Therapy | 8 (5%) |
| Research ethics | 6 (4%) |
| Real world evidence | 4 (3%) |
| Patient engagement in medicine development | 4 (3%) |
| Chemistry, manufacturing and controls | 3 (2%) |
| Law and governance | 3 (2%) |
| Outcomes methodology | 3 (2%) |
| Patient-reported outcomes | 3 (2%) |
| Vaccine | 3 (2%) |
| Drug discovery and toxicology | 2 (1%) |
| Health policy | 2 (1%) |
| Innovation and Research & Development | 2 (1%) |
| Virology | 2 (1%) |
| Drug metabolism and pharmacokinetic | 1 (0.6%) |
| Health and social care regulation | 1 (0.6%) |
| Law and regulatory governance | 1 (0.6%) |
| Pharmacology | 1 (0.6%) |
| Policy and evidence-based healthcare | 1 (0.6%) |

*Some participants specified more than one area of expertise
