# Supplementary material for: Advancing UK Regulatory Science Strategy in the Context of Global Regulation: a Stakeholder Survey
Source: Ther Innov Regul Sci. 2021 Feb 16;55(4):646–55. doi: 10.1007/s43441-021-00263-2 (PMC7885762; doi:10.1007/s43441-021-00263-2)
Supplement: Supplementary file 4 — Electronic supplementary material 4 (PDF 591 kb) [file 43441_2021_263_MOESM4_ESM.pdf]

Regulatory science - Individual response

1. Challenges and strategic areas of regulatory science in the UK

2. Demographics

2. Please select the main stakeholder group you belong to:

		Response Percent	Response Total
1	Healthcare professional	9.66%	14
2	Academic	17.93%	26
3	Patient representative	8.28%	12
4	Health technology assessment agency	3.45%	5
5	Regulator	11.72%	17
6	Pharmaceutical company	20.69%	30
7	Large med-tech	0.69%	1
8	Small or Medium Size Enterprise	6.21%	9
9	Ethicist	2.07%	3
10	Trade association (please state)	0.69%	1
11	Other (please specify):	18.62%	27
		answered	145
		skipped	0

Other (please specify): (27)

3. Approximately how many years experience do you have in the development or application of regulation

		Response Percent	Response Total
1	1 year or less	6.21%	9
2	2 - 5 years	25.52%	37
3	6 - 10 years	12.41%	18
4	More than 10 years	55.86%	81
		answered	145
		skipped	0

3. Defining regulatory science in healthcare

4. Below are presented different definitions of regulatory science. Which one do you think provides the most accurate definition based on your understanding of the discipline?				
			**Response Percent**	**Response Total**
1	"Regulatory Science is the science of developing new tools, standards, and approaches to assess the safety, efficacy, quality, and performance of all FDA-regulated products." (FDA, 2010)		11.03%	16
2	"Regulatory Science can be described as a range of scientific disciplines that are applied to the quality, safety and efficacy assessment of medicinal products that inform regulatory decision-making throughout the lifecycle of a medicine. It encompasses basic and applied medicinal science and social sciences and contributes to the development of regulatory standards and tools" (EMA, 2018)		49.66%	72
3	"The acquisition and analysis of data sufficient to inform decision making pertinent to the approval of safe and effective therapeutics, devices and cosmetics and ensuring the safety and nutritional value of the food supply." (FitzGerald, 2010)		3.45%	5
4	"The science of developing methods to quantitatively and/or qualitatively analyze and understand the causal relations and mechanisms of the substances and the phenomena around us, and measuring their positive and negative effects. Their efficacy and safety are appropriately predicted, evaluated and judged using the methods developed and the results obtained in the [regulatory science], contributing to public health." (The Pharmaceutical Society of Japan, 2016)		9.66%	14
5	"The application of the biological, medical and sociological sciences to enhance the development and regulation of medicines and devices in order to meet the appropriate standards of quality, safety and efficacy." (CASMI, 2013)		17.93%	26
6	Other (please specify):		8.28%	12
			answered	145
			skipped	0

4. Current challenges and opportunities in regulatory science

5. What are the most important features of future regulation for UK healthcare innovation, which regulatory sciences needs to more effectively enable. Select all that apply		Response Percent	Response Total
1	Data sharing	63.45%	92
2	Open access	27.59%	40
3	Costs associated to data sharing (e.g. the costs of anonymising data)	10.34%	15
4	Cooperation between agencies (e.g. MHRA, EMA and FDA)	82.07%	119
5	Dialogue/cooperation across stakeholders e.g. between academia – industry (including biopharma and med tech) – regulatory agencies – patients	75.86%	110
6	Remaining aligned with EU following the UK’s exit from the European Union	47.59%	69
7	Faster approval of medical products	46.21%	67
8	Development of a framework to make regulatory decisions about risks and benefits of products that increasingly involve new technology (e.g. - digitally-based products/AI and product development, production processes and novel supply chains) and target patient outcomes.	70.34%	102
9	Technological and scientific challenges -genomics and increased personalisation/specialisation of products	50.34%	73
10	Developing effective tools to track and evaluate outcomes for reimbursement	24.83%	36
11	Professional development (i.e. workshops or courses to supplement knowledge base)	20.00%	29
12	Workforce retention rates	8.97%	13
13	Other (please specify):	11.72%	17
		answered	145
		skipped	0

5. Current challenges and opportunities in regulatory science

6. Please rank (drag and drop the question boxes) the most important features of future regulation for UK healthcare innovation, which regulatory sciences needs to more effectively enable:		
**Item**	**Total Score [1]**	**Overall Rank**
Flexibility: the capability of regulations to adapt to novel products and target patient outcomes	663	1
Co-development: collaboration across sectors, e.g. patients, manufacturers, regulators and educators working together to develop appropriate training for novel product deployment	646	2
Responsiveness: the preparation of frameworks which enable timely innovation required by emerging events	639	3
Speed: the rate at which new products can reach the market	531	4
Reimbursement: developing effective tools to track and evaluate outcomes for "pay for performance" products	294	5
Education and professional development	272	6
[1] Score is a weighted calculation. Items ranked first are valued higher than the following ranks, the score is a sum of all weighted rank counts.	answered	145
	skipped	0

6. Strategic areas for development

7. Thinking about your stakeholder group, which of the actions below are most important for the development of UK regulatory science. Select all that apply Patient Centered Drug Development			
		**Response Percent**	**Response Total**
1	Ensure that patients' experiences, perspectives, needs, and priorities are captured and meaningfully incorporated into drug development and evaluation.	77.04%	104
2	Reinforce patient relevance in evidence generation, increased patient involvement in regulatory activities, including patient representatives as additional experts in Scientific Advisory Groups, as well as patient contribution to scientific advice and protocol assistance.	60.00%	81
3	Enhance understanding and appropriate use of methods to capture information on patient preferences and the potential acceptability of tradeoffs between treatment benefit and risk outcomes.	62.22%	84
4	Increase use and transparency of patient input as evidence in regulatory science decision-making.	57.78%	78

7. Thinking about your stakeholder group, which of the actions below are most important for the development of UK regulatory science. Select all that apply Patient Centered Drug Development				
			**Response Percent**	**Response Total**
5	Expand patient centered outcomes research by compiling datasets converted to standardized format across critical classes of drugs that are entered into the clinical trials repository.		34.81%	47
6	Identification and use of approaches and best practices to facilitate patient enrollment and minimizing the burden of patient participation in clinical trials.		53.33%	72
7	Other (please specify):		9.63%	13
			answered	135
			skipped	10

7. Strategic areas for development

8. Thinking about your stakeholder group, which of the actions below are most important for the development of UK regulatory science. Select all that apply Enhancing innovation				
			**Response Percent**	**Response Total**
1	Develop processes and maximise expertise in dealing with special/small populations, pragmatic trials and real world data		58.02%	76
2	Underpin cost effective research using real world data		41.98%	55
3	Develop/implement innovative methodologies and deliver comprehensive research to underpin standardization, control and innovation of biological medicines		34.35%	45
4	Explore further opportunities for reclassification of medicines		13.74%	18
5	Explore new technology projects on digital products, artificial intelligence/machine learning,		58.02%	76
6	Support the development of Advanced Therapies Medicinal Products (ATMPs) and novel biologicals by providing useful standards and all-stage expert scientific advice		45.04%	59

**8. Thinking about your stakeholder group, which of the actions below are most important for the development of UK regulatory science. Select all that apply Enhancing innovation**				
			**Response Percent**	**Response Total**
7	Develop new standards e.g. for biologicals including biosimilars, advanced therapies		33.59%	44
8	Support innovative strategies to reduce the use of antibiotics, e.g. implementation of novel vaccines and therapies		39.69%	52
9	Engage with the clinical diagnostics community to enhance reliability of disease diagnoses and commutability of results		37.40%	49
10	Explore innovative ways of using real world data to assess clinical effectiveness in routine clinical settings		65.65%	86
11	Explore developing more agile regulatory approvals processes for novel and generic products		60.31%	79
12	Explore developing standards for new areas e.g. digital health, artificial intelligence, machine learning		64.12%	84
13	Explore supporting opportunities in vaccines, combination products, software algorithms, remote site reporting and additives		38.17%	50
14	Other (please specify):		6.87%	9
			answered	131
			skipped	14

8. Strategic areas for development

**9. Thinking about your stakeholder group, which of the actions below are most important for the development of UK regulatory science. Select all that apply Proactive, robust surveillance**				
			**Response Percent**	**Response Total**
1	Develop systems and processes for integrated medicines and devices surveillance		54.40%	68
2	Optimise signal and and risk assessment functions to respond to risks in real time		60.00%	75
3	Develop professional expertise and systems to improve market surveillance of medical devices		40.80%	51

9. Thinking about your stakeholder group, which of the actions below are most important for the development of UK regulatory science. Select all that apply Proactive, robust surveillance				
			**Response Percent**	**Response Total**
	using the new regulations and its new data sets			
4	Develop and expand use of medical device electronic data standards with partners		40.00%	50
5	Encourage reporting of adverse incidents from patients and health care professionals		52.00%	65
6	Systematically evaluate effectiveness of risk minimisation and impact		47.20%	59
7	Work closely with key source countries to assure safe production and supply		24.80%	31
8	Enhance information sharing		56.00%	70
9	Enhance the role of pharmacovigilance in enabling the introduction of innovative medicines		50.40%	63
10	Other (please specify):		7.20%	9
			answered	125
			skipped	20

9. Strategic areas for development

10. Thinking about your stakeholder group, which of the actions below are most important for the development of UK regulatory science. Select all that apply Organisational excellence/efficiency				
			**Response Percent**	**Response Total**
1	Identify future capability needs and ensure the right skill mix is available to support innovation and deliver priority programmes and core functions		66.94%	83
2	Identify opportunities to recruit, retain and develop staff; including new training opportunities		40.32%	50
3	Invest in scientific capabilities to meet emerging needs. Invest in staffs specialist skill sets, and in facilities to deliver state of the art regulation and services.		70.16%	87
4	Focus on identifying and developing talent and on the importance of leadership, in the		46.77%	58

10. Thinking about your stakeholder group, which of the actions below are most important for the development of UK regulatory science. Select all that apply Organisational excellence/efficiency				
			Response Percent	Response Total
	context of organisational development in an environment of scientific advance and environmental change			
5	Explore new methodologies for detecting data integrity issues associated with regulatory studies		39.52%	49
6	Explore opportunities to develop collaborations and information sharing with key global regulators, international partnerships with WHO and other key players		74.19%	92
7	Other (please specify):		6.45%	8
			answered	124
			skipped	21

10. Current training and future needs

11. Due to its breadth and dynamism, regulatory sciences require life-long learning* and development. *Life-long learning: Voluntary and self-motivated education or learning, beyond formal education, to improve personal or professional development.				
			Response Percent	Response Total
1	Yes		95.16%	118
2	No		4.84%	6
			answered	124
			skipped	21

12. This learning and development needs to be responsive to the development of innovative technologies and products.				
			Response Percent	Response Total
1	Yes		99.19%	123
2	No		0.81%	1
			answered	124
			skipped	21

11. Current training and future needs

13. Are you aware of any regulatory science training courses or training schemes (programmes) in: Select all that apply

		Response Percent	Response Total
1	United Kingdom	45.16%	56
2	United States	25.81%	32
3	Any of the countries in the European Union	19.35%	24
4	Any other country	4.84%	6
5	I'm not aware of any of these training courses	39.52%	49
6	Other (please specify):	11.29%	14
		answered	124
		skipped	21

Other (please specify): (14)

1	29/04/2020 11:19 AM ID: 139746970	Questions 11 and 12 are too binary and don't allow for ""It depends..."" Question 10 isn't a question.
2	29/04/2020 14:26 PM ID: 139762116	I know that training is available for professionals at HRA and MHRA, and the NCRI has some designed by patients for patients. ECMC also offers training to professionals and to patients too (but we patients usually have problems getting anyone to fund us).
3	30/04/2020 17:24 PM ID: 139834816	Depends what we mean. There are relatively regular meetings held in Europe by EFGCP/EFPIA and also by CBI and DIA to discuss regulatory issues and to compare approaches eg EFGCP held a meeting on benefits and Risks of research, how do we address the balance. These are normally open to researchers and regulators. This usually gives people the opportunity to compare approaches across countries.<br>Other countries are interested in what the UK is doing, even within Europe.
4	06/05/2020 08:23 AM ID: 140217002	Pharma and Med Device organisations offer many.
5	11/05/2020 09:27 AM ID: 140269952	unsure what level of awareness is being queried
6	13/05/2020 09:32 AM ID: 140971387	I have previously attended GCP for Medical Devices training at UCL Joint Research Office, given by the company PharmExcel. However, I would like greatly increased awareness of training courses that provide relevant information for my stakeholder group.
7	13/05/2020 10:21 AM ID: 140973976	genuine regulatory science training is very limited. Much is badged as regulatory science but in reality is regulatory affairs - which is fundamentally not the same
8	14/05/2020 01:06 AM ID: 141067465	Tends to be in various forums. Regulatory forums...niche groups in US mainly.
9	15/05/2020 10:43 AM ID: 140278165	There are courses for various disciplines but no overarching courses that I am aware of
10	26/05/2020 12:13 PM ID: 141930881	I am aware of lots of courses on ethics and governance, scientific regulation and best practice schemes, but maybe this not quite my area.
11	27/05/2020 08:36 AM ID: 142005138	Life long learning is not just about courses. With respect to my answers to the previous set of questions, all researchers (whatever their interests) should engage in life-long learning and for many this is incorporated into their research practices. This is axiomatic
12	01/06/2020 17:01 PM ID: 141174474	not my area of expertise

13. Are you aware of any regulatory science training courses or training schemes (programmes) in: Select all that apply				
			Response Percent	Response Total
13	01/06/2020 22:26 PM ID: 142345917	EUPATI (European Patients Academy) has a module in its training course about regulatory science for patient advocates.		
14	10/06/2020 16:48 PM ID: 142900989	Not aware of a specific programme. Individual organisations run training on specific issues eg, MRC, HTA, NIHR, MHRA, HRA etc		

12. Current training and future needs

14. Have you attended any of these training courses before?				
			Response Percent	Response Total
1	Yes		33.06%	41
2	No		66.94%	83
			answered	124
			skipped	21

13. Current training and future needs

15. Please state the reasons why you haven't attended to any of these courses:			
		Response Percent	Response Total
1	Open-Ended Question	100.00%	85

14. Current training and future needs

16. Please state which courses you have attended and why:				
			Response Percent	Response Total
1	Open-Ended Question		100.00%	40
1	20/04/2020 15:13 PM ID: 139242506	Cskdmx		
2	23/04/2020 16:13 PM ID: 139395473	Kings College<br>Pharmaceutical Diploma training		

16. Please state which courses you have attended and why:

			Response Percent	Response Total
		Attended as part of higher medical training		
3	25/04/2020 09:36 AM<br>ID: 139332710	ICH GCP C0urse - Good Clinical Practice International Conference on Harmonisation of technical requirements for registration of pharmaceuticals for human use. I have developed new IMP's so needed to understand these directives.		
4	26/04/2020 08:06 AM<br>ID: 139499249	MSc Medical Technology quality and regulatory affairs - Sheffield Hallam University. I was the course leader!		
5	28/04/2020 20:05 PM<br>ID: 139717264	TOPRA workshop, Drugs Regulatory Affairs Module of Pharmaceutical Medicine Degree at King's College		
6	29/04/2020 12:59 PM<br>ID: 139755801	Required for my job<br>PATDD Cambridge https://www.thebts.org/events-2/courseandwebinars/patdd2020/<br>CE courses from SOT www.toxicology.org		
7	29/04/2020 14:26 PM<br>ID: 139762116	I have helped deliver training at MHRA and HRA and NCRI, and have received it at ECMC.		
8	29/04/2020 16:51 PM<br>ID: 139775581	Genomics and stem cells - use of. Our Ethics Committee would like to embark on this.		
9	30/04/2020 17:24 PM<br>ID: 139834816	Benefits and risks of research, how do we address the current imbalance? EFGCP<br>Risk adapted approaches to regulating health research - Kings College - this workshop was run by Annette Rid, see her work on this topic - she is now in the States.<br>These courses were relevant to me as it addressed for me the key issue. I am interested in how regulation can be made more proportionate to risk. The HRA is guilty of treating all research regardless of methodology as the same and using a sledgehammer to crack a nut. Research which was taken out of remit by GAfREC in 2011 eg studies using anonymised data now require review by the HRA for the research governance aspects of the work which is entirely pointless. The cost of the HRA exceeds its budget yet they continue to review studies that they do not need to see.<br>I have also taken part in numerous conferences on transparency and data disclosure run by CBI and UK. I can send you details if you are interested.<br>Also attended a course on Advanced Policy development but of course this is generic.		
10	03/05/2020 18:27 PM<br>ID: 140046656	FDA Clinical Outcome Assessments Workshops.		
11	05/05/2020 14:22 PM<br>ID: 140146212	Device regulations (Europe and USA)<br>Medical device clincial investigation design<br>Medical device regulatory audit<br><br>All relevant to my role as an external Notified Body clinical reviewer for medical devices		
12	05/05/2020 15:51 PM<br>ID: 140161374	ICH-GCP training		
13	05/05/2020 18:36 PM<br>ID: 140180370	AAADV		
14	06/05/2020 06:26 AM<br>ID: 140214899	Regulatory Science for Advanced (Gene and Cell) Therapy: Advanced Therapies - Bench to Medicine, UCL		
15	06/05/2020 17:37 PM<br>ID: 140293421	FDA Silversprings workshops		
16	07/05/2020 09:18 AM<br>ID: 140321234	courses with MHRA and industry organizations to keep up to date with emerging trends relevant for my area.		

16. Please state which courses you have attended and why:

			Response Percent	Response Total
17	13/05/2020 09:32 AM ID: 140971387	GCP for Medical Devices at UCL JRO, from PharmExcel		
18	14/05/2020 01:06 AM ID: 141067465	biotech and regulatory conferences		
19	14/05/2020 13:29 PM ID: 141111215	TOPRA		
20	14/05/2020 14:18 PM ID: 141117935	Free webinars for the purpose of self learning		
21	14/05/2020 14:53 PM ID: 141121846	Various and multiple pharmacovigilance training courses, other training courses in drug development and regulatory approval processes, GCP, CSV, etc.		
22	14/05/2020 14:57 PM ID: 141122636	Various.		
23	14/05/2020 16:38 PM ID: 141133519	PIPA PSMF course when starting a new role.		
24	14/05/2020 22:30 PM ID: 141145023	Courses run by the DSRU, in order to continue my own development		
25	15/05/2020 06:32 AM ID: 141166805	Kings' College/IFAPP Certificate in Medicines Development		
26	15/05/2020 13:29 PM ID: 141198429	FDA training courses as they come. FDA are a key stakeholder.		
27	19/05/2020 03:47 AM ID: 140019204	Am attending the Duke University 6 week Regulatory Affairs Training Progra, but also have attended Regulatory Science seminars through USC and others. I am interested in regulatory science through my work with the IDEAL Collaboration at Oxford, which is focused on complex interventions, including medical devices, innovation and evidence gathering in these, and in particular evidence gathering for Medical Devices. I am currently part of a working group and paper on evidence and regulation for implantable medical devices and another on using RWE in surgical and device innovation. This is an area I am very iterated in (My DPhil work is also centered on complex intervention clinical trials methods).		
28	19/05/2020 13:51 PM ID: 141557868	MHRA/HRA		
29	21/05/2020 14:48 PM ID: 141703952	Many programmes through TOPRA, DIA, RAPS and others, as well as universities in the UK and US. Why - see the article in the Journal of the Bleedin' Obvious.		
30	21/05/2020 15:25 PM ID: 141706963	TOPRA - high quality events with input from regulatory agencies		
31	21/05/2020 16:55 PM ID: 141716506	TOPRA modules, and EMA year end reviews		
32	21/05/2020 17:26 PM ID: 141719659	Online courses with Australian Universities: learning processes for ANZA regulatory of medicines<br>FDA training - med-dev / drug device learning<br>Attending RAPS and TOPRA courses.		
33	21/05/2020 17:44 PM ID: 141718643	I have attended and also presented on TOPRA courses in regulation of medical devices and IVD's to broaden my knowledge.		
34	23/05/2020 10:30 AM ID: 141824677	Boyd's Training on Medicines Regulation as part of ongoing learning and development.		
35	27/05/2020 09:06 AM ID: 141989381	TOPRA - directly relevant to role at the time, no internal exepertise to leverage		

16. Please state which courses you have attended and why:			
		Response Percent	Response Total
36	27/05/2020 14:30 PM<br>ID: 142033411	DSRU Postgrad diploma in pharmacovigilance<br>DIA QPPV Forum<br>EMA training days (when located in London)<br>PIPA conference<br>- for professional development, learning and to meet other people from the industry/ networking	
37	27/05/2020 16:17 PM<br>ID: 142043985	TOPRA, EMA, DIA	
38	27/05/2020 17:37 PM<br>ID: 142049718	Various TOPRA courses to enhance my regulatory knowledge.	
39	01/06/2020 22:26 PM<br>ID: 142345917	I was in the first cohort of EUPATI patient advocates about five years ago (and I was also co-chair of the EUPATI advisory board at the same time). I attended EUPATI because I wanted to hone my skills regarding medicines development. Even though I had already been a patient advocate for ten years, there were gaps in my knowledge, especially about some aspects of regulatory issues. I found it an extremely useful course.	
40	10/06/2020 16:48 PM<br>ID: 142900989	Our staff and members access short CPD sessions rather than training programmes. They will self-register. One big area of collaborative development currently is in Complex Innovative Design Trials where DHSC requested systematic development for health staff/research and volunteer members as part of sector skills development programme in 2019. There has been cross-organisation collaboration to deliver this. NIHR have had a programme of work resulting in eLearning and podcasts. The HRA has delivered learning to members at 5 regional events and worked with ECMC and others to produce 7 podcasts which will disseminated in alignment with those from NIHR.	
		answered	40
		skipped	105

15. Current training and future needs

17. What kind of additional/novel training formats and topics do you think regulatory science would benefit from?			
		Response Percent	Response Total
1	Open-Ended Question	100.00%	119
			119
		skipped	26

18. Would these training be useful for current or future regulatory science needs?			
		Response Percent	Response Total
1	For current priorities	78.99%	94

18. Would these training be useful for current or future regulatory science needs?				
			Response Percent	Response Total
2	For future priorities		84.87%	101
			answered	119
			skipped	26

16. Current training and future needs

19. At what level of education and training do you think regulatory science should be focused? Select all that apply				
			Response Percent	Response Total
1	Undergraduate		29.41%	35
2	Postgraduate - Taught MSc		62.18%	74
3	Postgraduate - Research MRes or PhD		52.10%	62
4	Academic fellowships		44.54%	53
5	Cross-sector fellowships		47.90%	57
6	Continuing Professional Development (short-course)		87.39%	104
			answered	119
			skipped	26

17. Infrastructure required

20. When working in a highly regulated area, do you know how to access expertise?				
			Response Percent	Response Total
1	Yes		84.87%	101
2	No		15.13%	18
			answered	119
			skipped	26

18. Infrastructure required

21. If you are to search for such expertise, would your starting point be the UK or international market?				
			**Response Percent**	**Response Total**
1	UK		84.03%	100
2	International market		21.85%	26
			answered	119
			skipped	26

19. Infrastructure required

22. Are you aware of the MHRA Innovation Office?				
			**Response Percent**	**Response Total**
1	Yes		76.47%	91
2	No		23.53%	28
			answered	119
			skipped	26

23. Have you accessed them?				
			**Response Percent**	**Response Total**
1	Yes		23.53%	28
2	No		76.47%	91
			answered	119
			skipped	26

20. Infrastructure required

24. From your experience, can you give examples of successful collaborations on health products development across sectors in the UK or internationally?			
		**Response Percent**	**Response Total**
1	Open-Ended Question	100.00%	116
		answered	116
		skipped	29

25. What elements made these a success?			
		**Response Percent**	**Response Total**
1	Open-Ended Question	100.00%	116

21. Infrastructure required

26. What legal, structural and other barriers have you experienced in creating collaborations?			
		**Response Percent**	**Response Total**
1	Open-Ended Question	100.00%	115

22. Infrastructure required

27. Do you see the need for academic leadership in the UK in developing cutting-edge technologies to evaluate safety, efficacy, quality, and performance of developed new health products and technologies?				
			**Response Percent**	**Response Total**
1	Yes		80.00%	92
2	No		20.00%	23
			answered	115
			skipped	30

23. Infrastructure required

28. Would you consider any of these area as particularly benefiting from academic leadership? Select all that apply				
			**Response Percent**	**Response Total**
1	Patient-centred technology development (including: patient reported outcomes, patient and public involvement and "rise of the small population")		64.04%	73
2	AI in healthcare		62.28%	71

**28. Would you consider any of these area as particularly benefiting from academic leadership? Select all that apply**				
			**Response Percent**	**Response Total**
3	Real World Evidence		64.91%	74
4	Accelerated access to medicines		24.56%	28
5	Innovative trial design		67.54%	77
6	Do you consider any other areas as particularly requiring academic input or leadership? Please specify		9.65%	11
7	Other (please specify):		25.44%	29
			answered	114
			skipped	31

24. Infrastructure required

**29. The US offers a range of fellowships in regulatory sciences developed in cooperation between academia, FDA and industry. Do you see such a need in the UK market?**				
			**Response Percent**	**Response Total**
1	Yes		85.96%	98
2	No		14.04%	16
			answered	114
			skipped	31

25. Additional information

**30. Do you have any other comments you would like to add?**			
		**Response Percent**	**Response Total**
1	Open-Ended Question	100.00%	53

27. Additional information

32. Would you like to be acknowledged in future publications?				
			Response Percent	Response Total
1	Yes		35.96%	41
2	No		64.04%	73
			answered	114
			skipped	31
